# Hybrid Nanopore-Illumina Genome Assembly of a Drosophila suzukii Gut Bacterial Symbiont, Gluconobacter cerinus FLW-1

**DOI:** 10.1128/MRA.00190-21

**Published:** 2021-04-22

**Authors:** Runhang Shu, Adam C. N. Wong

**Affiliations:** a Entomology and Nematology Department, University of Florida, Gainesville, Florida, USA; b Genetics Institute, University of Florida, Gainesville, Florida, USA; Indiana University, Bloomington

## Abstract

*Gluconobacter* is a genus of acetic acid bacteria (AAB) whose members have been shown to function as insect symbionts. Here, we report the complete genome sequence of Gluconobacter cerinus, isolated from field-collected Drosophila suzukii using a hybrid assembly approach. The data provide essential insights into the metabolic functions of the symbiont to the host.

## ANNOUNCEMENT

The bacterial genus *Gluconobacter* (family *Acetobacteraceae*) is a member of the acetic acid bacteria (AAB) group. Recent research has highlighted AAB as widespread symbionts in insects, particularly insects of the orders Diptera, Hymenoptera, and Hemiptera (1). For example, Gluconobacter cerinus has been isolated from the ovaries of the oriental fruit fly (Bactrocera dorsalis) and shown to support host egg production (2). Recently, we isolated a *G. cerinus* strain from Drosophila suzukii specimens collected on a blackberry farm in Hawthorne, FL. Here, we present the complete genome sequence of *G. cerinus*, obtained using a hybrid assembly pipeline integrating sequences obtained from the Illumina and Oxford Nanopore Technologies (ONT) platforms.

The whole guts of *D. suzukii* were dissected and homogenized in phosphate-buffered saline (PBS). The homogenate was plated onto De Man, Rogosa and Sharpe (MRS) agar and incubated at 37°C for 48 h. Bacterial colonies were picked and grown in MRS broth overnight before being pelleted by centrifugation. Total genomic DNA was isolated using a phenol-chloroform protocol described previously (3). DNA for Illumina sequencing was fragmented by sonication, end polished, and ligated with the adapters; this was followed by PCR amplification and purification using the NEBNext Ultra II kit (New England Biolabs). The products were sequenced using the Illumina NovaSeq 6000 platform. The genomic library for ONT sequencing was prepared using the ligation sequencing kit 1D (SQK-LSK109) as per the manufacturer’s instructions. The genomic DNA was neither size selected nor sheared. ONT sequencing was performed on an R9.4.1 flow cell in a MinION sequencer. Real-time local base calling by Guppy was completed using MinKNOW v20.10.3 software.

The Illumina sequencing generated 9,593,212 reads with a total of 719 Mbp, while the MinION sequencer generated 2,634,889 reads, accounting for 7,851 million bases with a median read length of 2,877 bases. The longest read was 496,942 bases, and the read length *N*_50_ value was 3,945 bases. The low-quality reads and adapters of the Illumina sequences were removed using Trimmomatic v0.39 (4). The adapters and chimeric sequences of the Nanopore reads were removed using Porechop v0.2.4, followed by (i) filtering reads shorter than 2,000 bp, (ii) keeping the top 90% high-quality reads determined by 16-mers in Illumina reads, and (iii) subsetting 1,500 Mbp for the hybrid assembly using Filtlong v0.2. Finally, the Illumina and Nanopore reads, accounting for over 600× coverage, were aligned using Unicycler v0.4.8 (5). The assembly graphs or contigs were visualized in Bandage v0.8.1 (6). The assembled genome sequence has a total length of 3,328,849 bp, consisting of four contigs, with the first and largest contig making up 96% of the assembly. The genome was annotated in NCBI PGAP v5.0 (7) and PATRIC v3.6.8 (8). The PGAP annotation identified 2,938 coding DNA sequences and 79 RNA sequences for the largest contig (60 tRNAs, 15 rRNAs, and 4 noncoding RNAs [ncRNAs]). PATRIC indicates 97.3% fine consistency, 100% completeness, and 0.6% contamination.

### Data availability.

This whole-genome shotgun project has been deposited at DDBJ/ENA/GenBank under the accession number JAFEJB000000000. The version described in this paper is JAFEJB010000000. The raw sequencing reads have been deposited in the NCBI Sequence Read Archive (SRA) under the accession numbers SRR13680735 (ONT) and SRR13680736 (Illumina).
